# Supplementary material for: Extremely large magnetoresistance in few-layer graphene/boron–nitride heterostructures
Source: Nat Commun. 2015 Sep 21;6:8337. doi: 10.1038/ncomms9337 (PMC4595716; doi:10.1038/ncomms9337)
Supplement: Supplementary Information — Supplementary Figures 1-11 and Supplementary Notes 1-2 [file ncomms9337-s1.pdf]

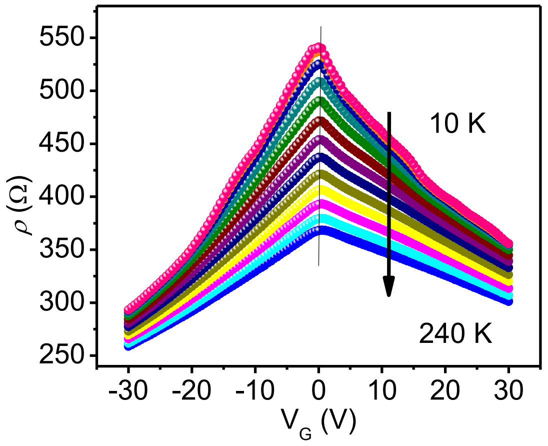


**Supplementary Figure 1. Resistivity from few-layer graphene on $SiO_2$.** Resistivity ($\rho$) as a function of back gate voltage ($V_G$) at various temperatures from 10 to 240 K. The results are from exfoliated few-layer graphene (< 10 layers) samples on $SiO_2$ substrate. The lithographically patterned sample has been annealed for 1 hour at 400 K under high vacuum conditions prior to the measurements. The charge neutrality point (maximum resistivity point) is seen at a gate voltage of ~ 0 V.

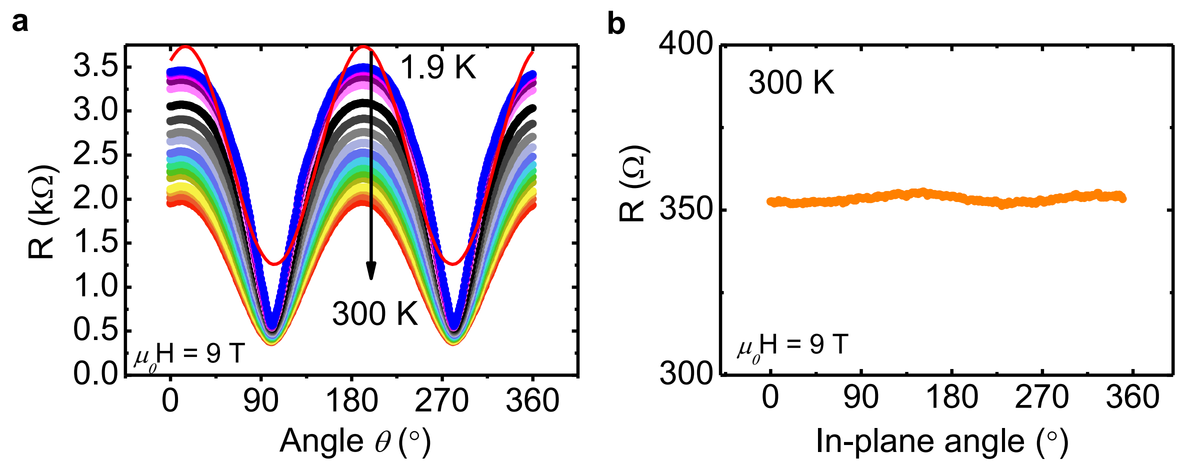


**Supplementary Figure 2**. **Angular magnetoresistance**. (**a**) The resistance vs. out-of-plane field angle. Here $\theta$ = 0 and 90° indicates the magnetic field is perpendicular and parallel to the sample plane, respectively. At 300 K, the MR can be fitted with a cosine function (similar to that reported in the main text), however as the temperature decreases from 300 K, the MR deviates from a cosine dependence (cosine fit for 2 K data is shown as solid red curve). Additional contribution to the MR is probably arising from quantum and the Zeeman spin Hall effects. (**b**) The resistance as a function of the angle between the current and in-plane magnetic field, clearly suggesting a negligible contribution of the interlayer interactions to the total MR. The angle between the magnetic field and current in the plane of the sample gives rise to only ~1% correction to the MR. The geometry related MR correction is seen as a difference in the maximum MR (minimum MR) which is < 0.5%.

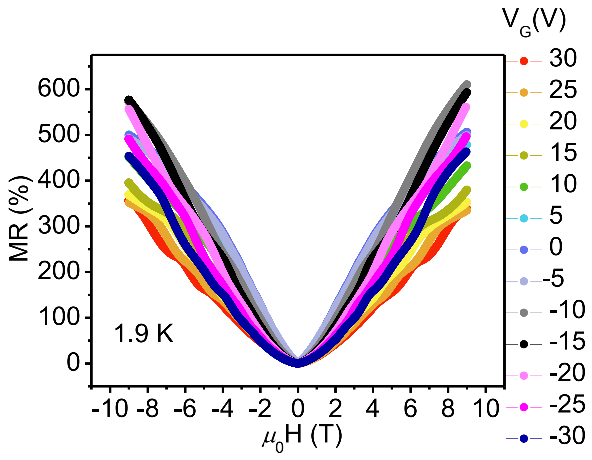


**Supplementary Figure 3. Gate voltage dependent magnetoresistance.** The MR vs. external magnetic field (*H*) as a function of back gate voltage ($V_G$) at 1.9 K. Oscillations are seen which is a result of partial quantization of the orbitals due to the 2D character of the charged particles, however the MR correction due to this quantum effect is small. The MR is gate tunable suggesting that the intrinsic nature of graphene is playing an important role.

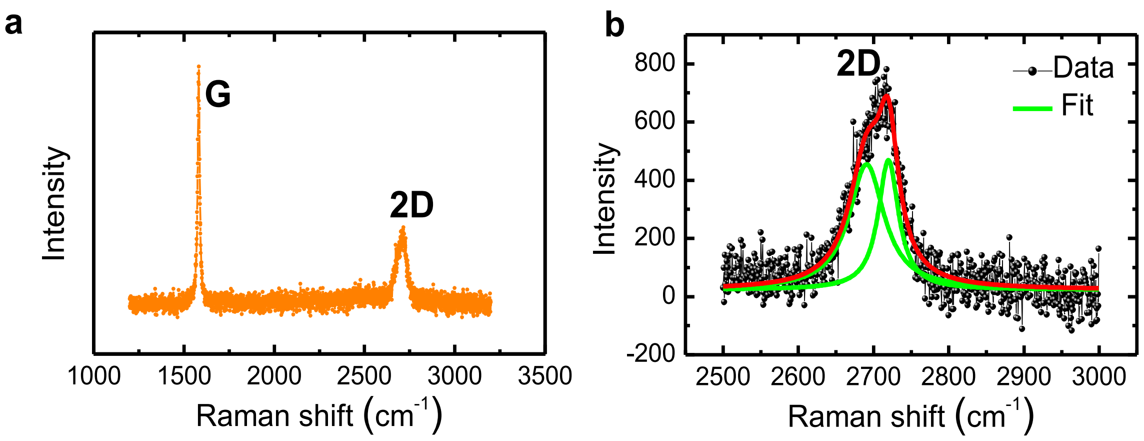


**Supplementary Figure 4. Raman spectrum of few-layer graphene**. (**a**) Raman spectrum of 6 layer graphene reported in the main text showing the characteristic modes with no disorder peak D. (**b**) Fit of 2D peak with two Lorentians, suggesting a few-layer graphene with ~ 6 layers which has been verified by atomic force microscopy (AFM) measurements.

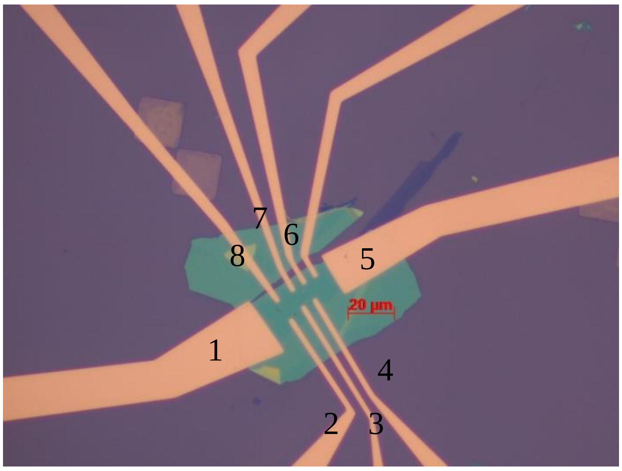


**Supplementary Figure 5. Device image of the van der Pauw sample.** Optical image of 6 layer graphene/BN sample used to measure in van der Pauw geometry which is described in the main text. Width (*W*) of the wire is ~ 20 μm. The current is passed between electrodes 1 and 2 and the voltage is measured between 3 and 5 with an electrode separation (*L*) of 7 μm and a *L/W* ratio of ~0.35.

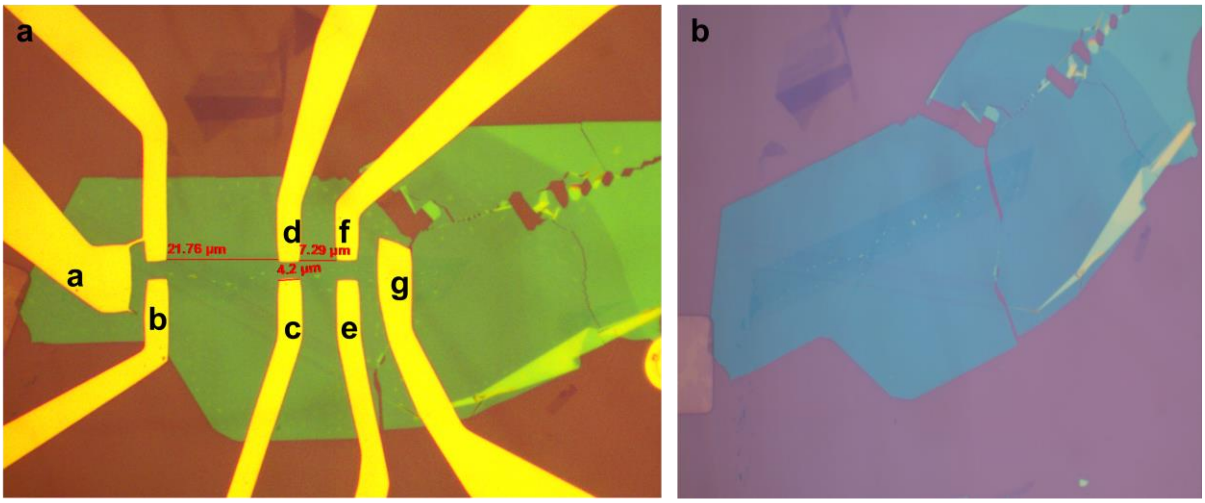


**Supplementary Figure 6. Device image of the 4 layer graphene on BN utilized in non-local measurements.** (**a**) Optical image of the lithographically patterned 4 layer graphene on BN. Graphene has been selected to have a smaller channel width to confine the current flow. This has been done to separate various contributions such as ohmic, thermal, and spins to the total MR. (**b**) Enlarged image with a better contrast of 4 layer graphene on BN. The non-local signal measurements have been carried out at two separations ($L$) of 21 and 29 μm from the current source electrodes. The width ($W$) of the graphene channel is 7 μm. The ohmic contribution to the measured non-local signal varies as exp-($\pi L/W$) and a $L/W$ ratio of ~3 is considered to be good enough to have negligible ohmic contribution to the total non-local signal.

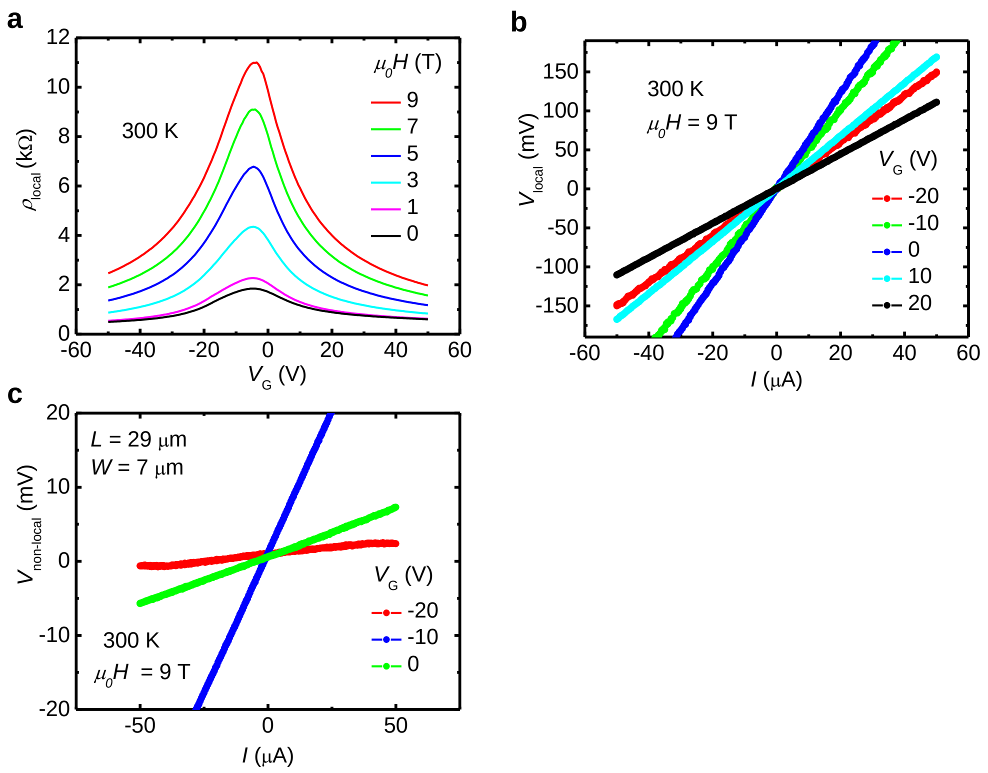


**Supplementary Figure 7. Non-local transport measurements at $L$ = 29 μm.** (**a**) The gate voltage ($V_G$) dependent channel resistivity ($\rho$) at 300 K as a function of an applied magnetic field normal to the graphene plane in the local geometry. The charge neutrality point (CNP) is close to zero. A large positive magnetoresistance (MR) is seen at all gate voltages, however the MR is maximum at the CNP. (**b**) The local current ($I$) - voltage ($V$) characteristic curves as a function of $V_G$ at 300 K and with a normal magnetic field of 9 T. The linear $I$-$V$ suggests an ohmic contact between Cr/Au electrodes and graphene. (**c**) The non-local $I$-$V$ characteristic curves as a function of gate voltage and at a normal magnetic field of 9 T and 300 K. The linear relation between the non-local voltage and local charge current suggests the Ettingshausen – Nernst effect as the possible cause. The magnitude of the non-local voltage is largest at the CNP and decays rapidly on both sides of the CNP. The non-local signal is very weak in the absence of a magnetic field implying little contribution from the spin Hall effect. In addition, due to carbon's low atomic number, the possibility of atomic spin-orbit interaction contributing to the spin Hall effect is very unlikely.

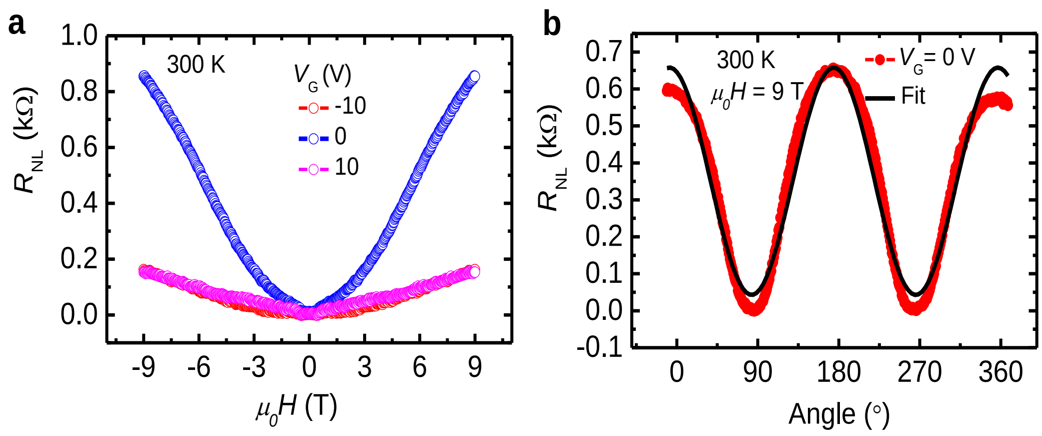


**Supplementary Figure 8. Room temperature non-local magnetoresistance.** (**a**) The magnetoresistance at 300 K as a function of back gate voltage ($V_G$) in the non-local geometry. The magnetic field is normal to the graphene plane. The MR is positive, giant and maximum at the CNP for unit applied magnetic field. The MR decays very rapidly on both sides of the CNP, suggesting that the non-local signal is maximum at the CNP. (**b**) Angle dependent non-local resistance ($R_{NL}$) at a magnetic field of 9 T and 300 K. MR follows a cosine dependence with the angle of the magnetic field wherein the sample exhibits a maximum MR, when the magnetic field is normal to the graphene plane (angle $\theta$ = 0° and 180°), and a minimum when the magnetic field is in the plane (angle $\theta$ = 90° and 270°). The MR variation with angle in the non-local geometry is very similar to that of local geometry, indicating a connection between them. However, unlike the local MR, the non-local MR is seen only close to the CNP.

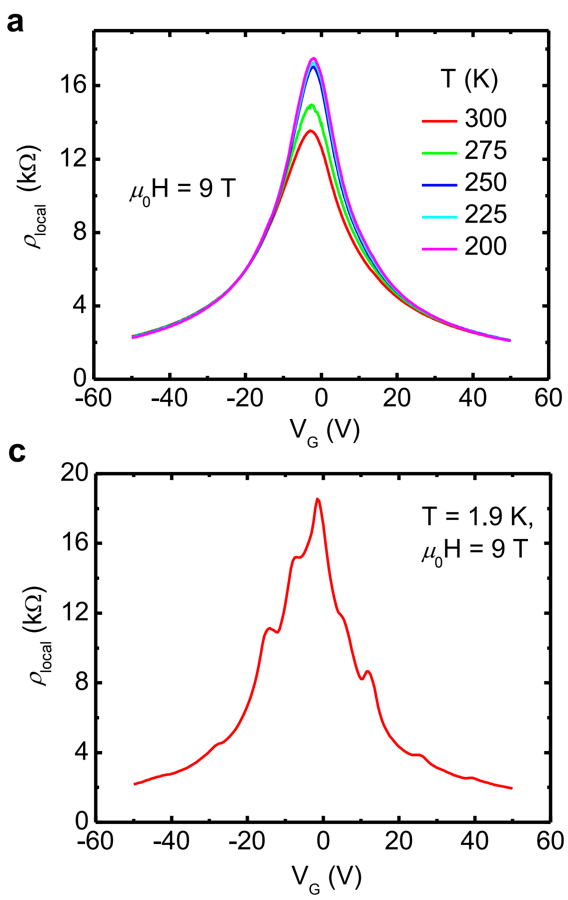


**Supplementary Figure 9. Temperature dependent magnetoresistance.** (**a**) The gate voltage ($V_G$) dependent channel resistivity ($\rho$) as a function of temperature in the local geometry. A normal magnetic field of 9 T is applied. (**b**) The gate voltage ($V_G$) dependent non-local resistance ($R_{NL}$) as a function of temperature. A normal magnetic field of 9 T is applied. (**c**) The gate voltage ($V_G$) dependent channel resistivity ($\rho$) at 1.9 K and a normal magnetic field of 9 T in the local geometry. The Shubnikov–de Haas (SdH) oscillations are seen in the resistivity due to Landau level quantization. (**d**) The gate voltage ($V_G$) dependent non-local resistance ($R_{NL}$) at 1.9 K in the non-local geometry. SdH oscillations are also seen in the non-local resistance with a large increase in the resistance at the CNP. The large increase in the non-local signal at low temperatures can be explained based on an increase in accumulation of the charge carriers at the non-local contacts due to weaker lattice scattering, which increases the thermal gradient.

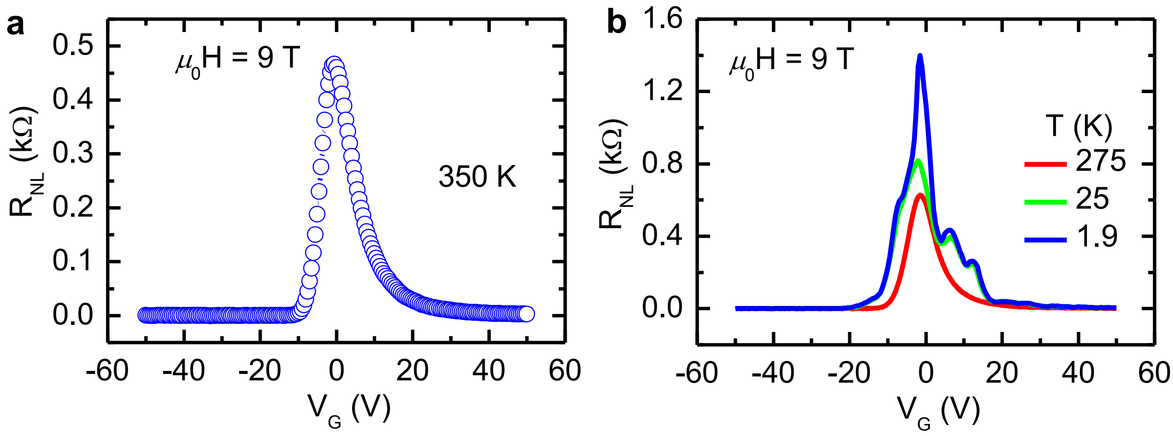


**Supplementary Figure 10. Non-local transport measurement at *L* = 21 μm.** Non-local resistance ($R_{NL}$) as a function of back gate voltage ($V_G$) at a temperature of (**a**) 350 K, (**b**) 275 K, 25 K and 1.9 K. The non-local electrodes are separated by 21 μm from the current source electrodes. The large non-local voltage is seen only near the CNP in contrast to the local resistivity. It is interesting to note that the non-local voltage persists at a temperature of 350 K implying its potential in practical applications. At low temperatures, Shubnikov-de Haas oscillations are seen in the non-local geometry with a giant increase in the non-local signal at the CNP.

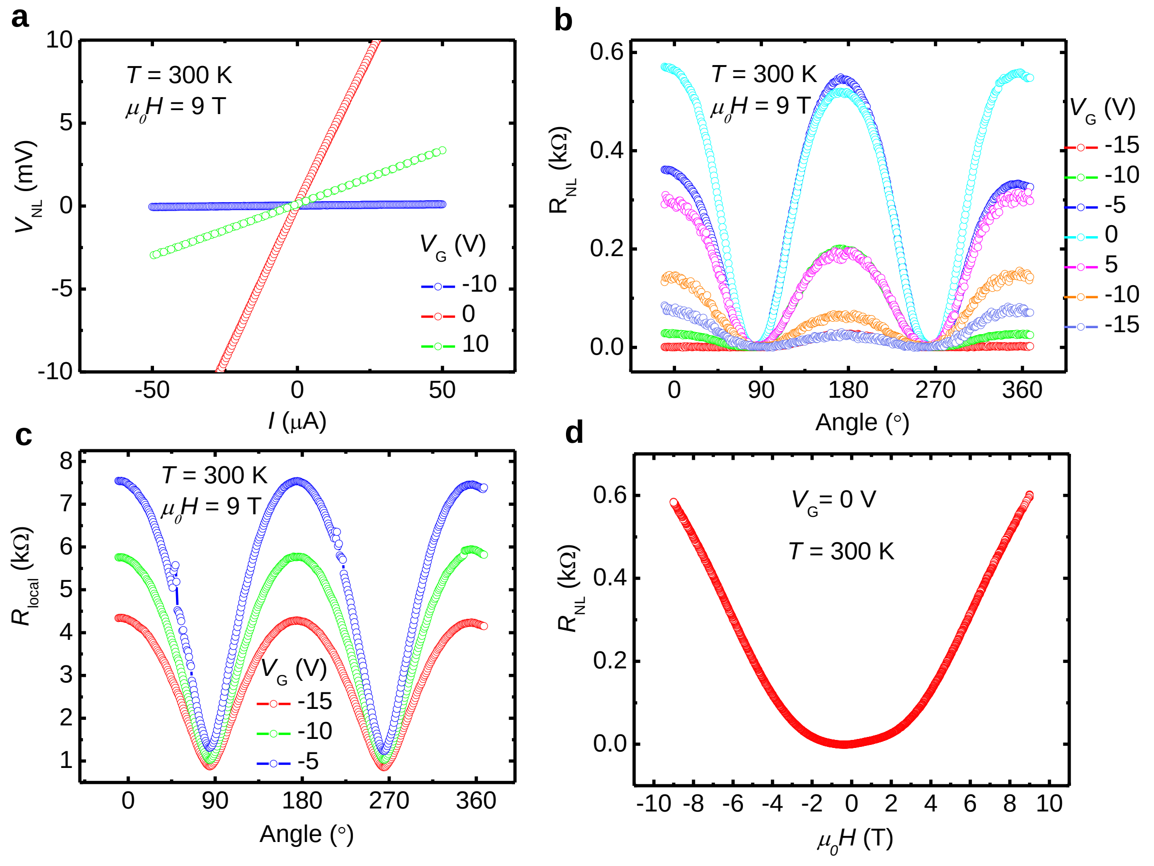


**Supplementary Figure 11. Angular local and non-local magnetoresistance.** (**a**) The non-local current ($I$) - voltage ($V$) characteristic curves as a function of $V_G$ at 300 K and a normal magnetic field of 9 T. The non-local electrodes are separated by 21 μm from the current source electrodes. It is evident that there is a finite non-local voltage at 300 K, which is a linear function of the local charge current. The linear relation between the non-local voltage and local charge current suggests the Ettingshausen – Nernst effect as the possible cause. The magnitude of the non-local voltage is largest at the CNP and decays rapidly on both sides of the CNP. (**b**) The angle dependent non-local MR as a function of $V_G$ at 300 K. Here $\theta = 0°$ means the magnetic field is normal to the graphene plane and $\theta = 90°$ means the magnetic field is parallel to the plane. It is seen that the non-local MR is maximum when the magnetic field is normal to the sample plane. At the CNP, the maximum value of MR is nearly symmetric for angles 0, 180 and 360 degrees, implying minimum contribution from geometry. However, for $V_G$ different from CNP, the magnitude of the maxima in the MR

shows non-symmetric characteristics, implying contributions from a geometry effect, which provides an opportunity to understand the geometry effects on the non-local MR and its tunability. (**c**) In order to see if the local MR has any geometry dependence, angle dependent MR measurements have been carried out as a function of $V_G$ at 300 K and 9 T. The MR varies as cos$\theta$. The maximum value of the MR is symmetric for various angles (0, 180 and 360°) implying negligible contribution from geometry to the local MR. (**d**) To further understand the nature of the non-local MR at a separation of 21 μm, the non-local MR measurements have been carried out at 300 K and zero gate bias. The MR is positive with a very large magnitude at a normal magnetic field of 9 T.

**Supplementary Note 1. Two-channel model**

With the aim of achieving and optimizing a large magnetoresistance (MR) in layered two dimensional (2D) materials, especially at low magnetic fields, we model the transport behavior of few layer graphene in the presence of external magnetic and electric fields. We consider two 2D films under a common transverse magnetic field (**B**) and a longitudinal electric field (**E**). Assume that each channel has a three dimensional (3D) density $n_{i=1,2}$ of carriers and a charge mobility $\mu_{i=1,2}$, and both obey the Drude model for conduction so that in the absence of a magnetic field, its conductivity is given by $\sigma_{D,i} = en_i\mu_i$. In the presence of a transverse magnetic field the 3D current density is given by $\mathbf{j}_i = \sigma_i.\mathbf{E}$, where $\sigma_i = \begin{bmatrix} \sigma_{xx,i} & \sigma_{xy,i} \\ \sigma_{yx,i} & \sigma_{yy,i} \end{bmatrix}$ is the conductivity tensor. In the Drude model it is easy to show that

$$\sigma_{xx,i} = \sigma_{yy,i} = \frac{\sigma_{D,i}}{1+(B\mu_i)^2} = \frac{en_i\mu_i}{1+(B\mu_i)^2} \tag{1}$$

$$\sigma_{xy,i} = \sigma_{yx,i} = \frac{B\mu_i\sigma_{D,i}}{1+(B\mu_i)^2} = \frac{eBn_i\mu_i^2}{1+(B\mu_i)^2} \ . \tag{2}$$

If we put two channels together and since they are under the same electric fields, the total current running through the stack will be $\mathbf{J} = \mathbf{j}_1+\mathbf{j}_2 = (\sigma_1+\sigma_2)\mathbf{E}$. The total conductivity of the sample is $\sigma = \sigma_1+\sigma_2$, which is nothing but the condition of two resistors in parallel. The only difference is that now we have sum of two tensors, instead of scalars. The resistivity tensor is obtained by inversion of the conductivity tensor: $\rho = \sigma^{-1}$ and gives

$$\rho_{xx} = \rho_{yy} = \frac{\sigma_{xx}}{\sigma_{xx}^2 + \sigma_{xy}^2} \tag{3}$$

$$\rho_{xy} = -\rho_{yx} = \frac{\sigma_{xy}}{\sigma_{xx}^2 + \sigma_{xy}^2} \ . \tag{4}$$

We find that the resistivity of the stack is given by

$$e\rho_{xx} = \frac{\frac{n_1\mu_1}{1+B^2\mu_1^2} + \frac{n_2\mu_2}{1+B^2\mu_2^2}}{\left(\frac{n_1\mu_1}{1+B^2\mu_1^2} + \frac{n_2\mu_2}{1+B^2\mu_2^2}\right)^2 + B^2\left(\frac{n_1\mu_1{}^2}{1+B^2\mu_1^2} + \frac{n_2\mu_2{}^2}{1+B^2\mu_2^2}\right)^2} \tag{5}$$

$$e\rho_{xy} = B\frac{\frac{n_1\mu_1{}^2}{1+B^2\mu_1^2} + \frac{n_2\mu_2{}^2}{1+B^2\mu_2^2}}{\left(\frac{n_1\mu_1}{1+B^2\mu_1^2} + \frac{n_2\mu_2}{1+B^2\mu_2^2}\right)^2 + B^2\left(\frac{n_1\mu_1{}^2}{1+B^2\mu_1^2} + \frac{n_2\mu_2{}^2}{1+B^2\mu_2^2}\right)^2}. \tag{6}$$

There are two obvious limits, case 1: $B = 0$ in which we get the trivial result;

$$\rho_{xx,0} = \frac{1}{e(n_1\mu_1 + n_2\mu_2)}, \tag{7}$$

$$\rho_{xy} = 0. \tag{8}$$

Notice that in this case if $n_1\mu_1 >> n_2\mu_2$ we get $\rho_{xx,0} = 1/(en_1\mu_1)$ indicating that the resistivity is determined by the mobility of the most conducting channel, in this case channel 1.

In the opposite limit of case 1 where $B >> \mu_1^{-1}, \mu_2^{-1}$ we find

$$\rho_{xx,\infty} = \frac{n_1\mu_2 + n_2\mu_1}{e\mu_1\mu_2(n_1 + n_2)}, \tag{9}$$

$$\rho_{xy,\infty} \approx \frac{B}{e(n_1 + n_2)}. \tag{10}$$

Note that the Hall resistance is what one would expect in a large magnetic field, i.e., it depends only on the carrier concentration. However, unlike the case of zero field, if the two channels are such that $n_2\mu_1 >> n_1\mu_2$ we have

$$\rho_{xx,\infty} \approx \frac{n_2}{n_1 + n_2}\frac{1}{\mu_2} \tag{11}$$

and the resistivity is determined by the mobility of the least conducting channel, in this case channel 2. Note that the result indicates that the maximum MR is given by

$$MR_\infty = \frac{\rho_{xx,\infty} - \rho_{xx,0}}{\rho_{xx,0}} = \frac{n_1 n_2}{(n_1 + n_2)^2} \frac{(\mu_1 - \mu_2)^2}{\mu_1 \mu_2} \,. \tag{12}$$

Thus in the case where $\mu_1 >> \mu_2$ we find

$$MR_\infty \approx \frac{n_1 n_2}{(n_1 + n_2)^2} \frac{\mu_1}{\mu_2} >> 1 \tag{13}$$

and the MR can be arbitrarily large.

**Supplementary Note 2. Multi-channel model**

The above considerations can be generalized for any number of channels, N. Once again, the main assumption is that each channel obeys the Drude model and that the resistances add in parallel. It can be shown that

$$e\rho_{xx} = \frac{\sum_{i=1}^{N} \frac{n_i \mu_i}{1+B^2\mu_i^2}}{\left(\sum_{i=1}^{N} \frac{n_i \mu_i}{1+B^2\mu_i^2}\right)^2 + B^2\left(\sum_{i=1}^{N} \frac{n_i \mu_i^2}{1+B^2\mu_i^2}\right)^2} \tag{14}$$

$$e\rho_{xy} = B\frac{\sum_{i=1}^{N} \frac{n_i \mu_i^2}{1+B^2\mu_i^2}}{\left(\sum_{i=1}^{N} \frac{n_i \mu_i}{1+B^2\mu_i^2}\right)^2 + B^2\left(\sum_{i=1}^{N} \frac{n_i \mu_i^2}{1+B^2\mu_i^2}\right)^2}. \tag{15}$$

In weak magnetic fields, $B << \mu^{-1}$, we find

$$\rho_{xx} \approx \rho_0 + eB^2\rho^2[\sum_i n_i\mu_i^3 - e\rho_0(\sum_i n_i\mu_i^2)^2], \quad \rho_0 = \frac{1}{e\sum_i n_i\mu_i}. \tag{16}$$

In strong magnetic fields, $B >> \mu^{-1}$, we find

$$\rho_{xx} \approx \rho_\infty[1-\frac{1}{B^2}(\frac{\sum_i n_i\mu_i^{-3}}{\sum_i n_i\mu_i^{-1}} - 2\frac{\sum_i n_i\mu_i^{-2}}{\sum_i n_i} + \frac{(\sum_i n_i\mu_i^{-1})^2}{(\sum_i n_i)^2}], \quad \rho_\infty = \frac{\sum_i n_i\mu_i^{-1}}{(\sum_i n_i)^2}. \tag{17}$$

Hence the maximum magnetoresistance is given by

$$MR_\infty = \frac{\rho_\infty - \rho_0}{\rho_0} = \frac{\sum_i n_i\mu_i}{\sum_i n_i}\frac{\sum_i n_i\mu_i^{-1}}{\sum_i n_i} - 1. \tag{18}$$
